# Sperm whale clans and human societies

**DOI:** 10.1098/rsos.231353

**Published:** 2024-01-10

**Authors:** Hal Whitehead

**Affiliations:** Biology Department, Dalhousie University, Halifax, Nova Scotia, Canada B3H 4R2

**Keywords:** sperm whale, clan, ethno-linguistic group, culture, large-scale societies, symbolic marker

## Abstract

Sperm whale society is structured into clans that are primarily distinguished by vocal dialects, which may be symbolic markers of clan identity. However, clans also differ in non-vocal behaviour. These distinctive behaviours, as well as clan membership itself, are learned socially, largely within matrilines. The clans can contain thousands of whales and span thousands of kilometres. Two or more clans typically use an area, but the whales only socialize with members of their own clan. In many respects the closest parallel may be the ethno-linguistic groups of humans. Patterns and processes of human prehistory that may be instructive in studying sperm whale clans include: the extreme variability of human societies; no clear link between modes of resource acquisition and social structure; that patterns of vocalizations may not map well onto other behavioural distinctions; and that interacting societies may deliberately distinguish their behaviour (schismogenesis). Conversely, while the two species and their societies are very different, the existence of very large-scale social structures in both sperm whales and humans supports some primary drivers of the phenomenon that are common to both species (such as cognition, cooperation, culture and mobility) and contraindicates others (e.g. tool-making and syntactic language).

## 1. Introduction

We are fascinated by the history of humanity: how societies grew, developed structure and sometimes collapsed; how these societies interacted with the natural world and with each other; how they developed and used technologies; how the humans within groups of very different sizes were or were not organized; and how, eventually, we ended up with the world around us [1–3]. In their 2021 book ‘The Dawn of Everything’, David Graeber and David Wengrow present a ‘new history of humanity’, making a largely convincing case that much of what we thought we knew about the nature, dynamics and history of human sociocultural groupings was either simplistic, badly incomplete or wrong [4]. Some have disputed the apparent political implications and some specific archaeological interpretations in ‘The Dawn of Everything’ [e.g. 5] but the authors' general picture of the complex dynamics of human societies is convincing. Humanity's history is very involved.

My reading of ‘The Dawn of Everything’ suggested some parallels between the structures and dynamics of the large-scale social structures of the species with the largest brain relative to body size (humans*; Homo sapiens*) and that with largest brain (sperm whales; *Physeter macrocephalus*) [6]. These parallels, and their implications, are the focus of this paper.

The sperm whale is an animal of extremes [7]. It is the most phylogenetically divergent species of all extant cetaceans (i.e. whales, dolphins and porpoises) [8]. Its large body is dominated by the largest nose in the animal world, which contains the spermaceti organ. The spermaceti organ primarily functions as a sonar, making clicks with the highest sound pressure level of any animal [9], allowing sperm whales to find their deep-water prey efficiently [10]. The spermaceti organ contains a particularly fine grade of oil. Consequently, the sperm whales were a prime target of whalers, and over a million of them were killed across the deep oceans between 1712 and 1982 [11].

With the end of whaling, the emphasis shifted to studying the living animals, including their social lives. Sperm whales, and in particular the females and young, are clearly very social, almost always seen close to one another, often gathering and rolling around each other for minutes to hours at the surface, and forming important life-long bonds [12,13]. Apparent parallels between the large-scale social organization of humans with that of a species living in a very different habitat may help us understand the societies of sperm whales, particularly their clans, and indicate processes of social evolution that are not purely products of human exceptionalism.

The goals of this paper are: to summarize what is known of sperm whale clans, highlighting some unexpected findings; to compare and contrast the clans with human ethno-linguistic groups; to consider how new knowledge of the nature and dynamics of human ethno-linguistic groups can provide guidance for ongoing research into sperm whale clans; and to suggest how the existence of sperm whale clans may have implications for understanding human social evolution at the largest scales.

## 2. Sperm whale clans

The foundation of sperm whale society is the matrilineally based social unit of ten or so females and their offspring. The members of the unit travel together, suckle each others' infants, and babysit them while mothers make long deep dives to feed [14,15]. They also defend themselves communally against their only serious natural predator, the killer whale (*Orcinus orca*) [16]. In contrast to many terrestrial mammals, intraspecific contest competition for food or space is unlikely to be significant among sperm whales in their deep ocean habitat, so that cooperative care, communal defence against predators and knowledge sharing seem to be the principal benefits of sociality [7]. Male sperm whales leave their natal social units in their teens and head to higher latitudes, away from their mothers and other female relatives who remain in warm waters [17]. This gives the sperm whale another extreme: the greatest sexual segregation of any species.

However, when encountered at sea there are often more than 10 animals present. The units form groups with other units for periods of hours to days [18]. The groups are not random. A social unit has some social units that it will often group with, and others, which use the same geographical area, with which it does not group [13,18].

**Figure 1.**
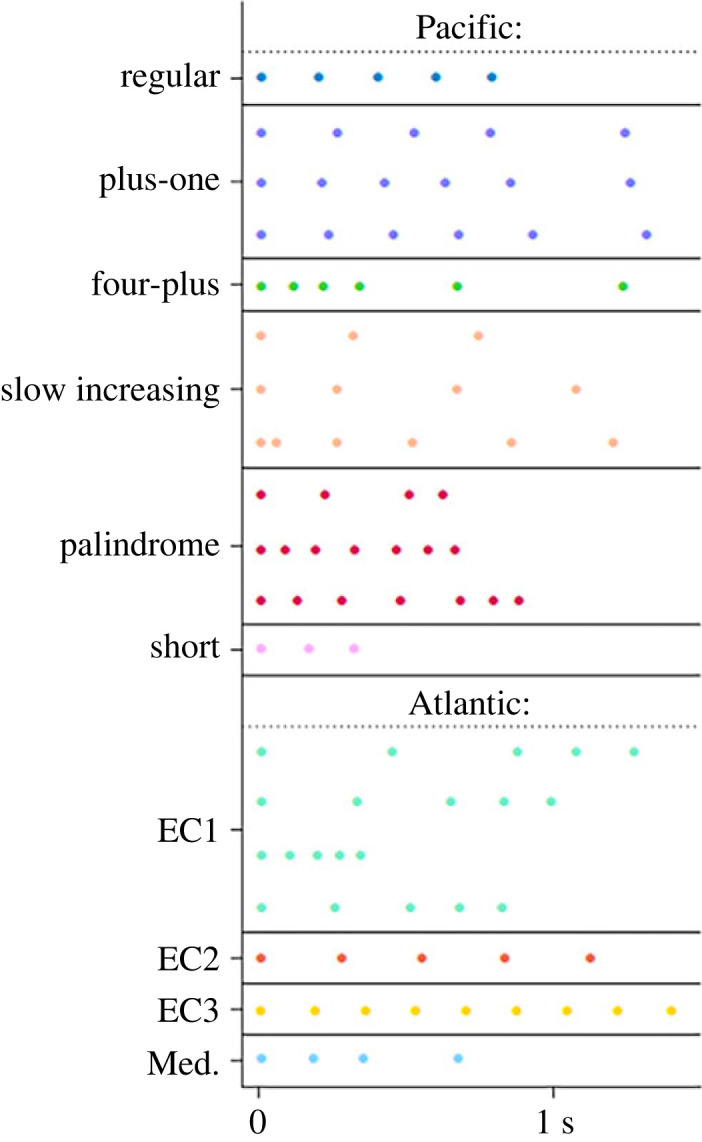
Identity codas (stereotypical patterns of clicks made primarily by one clan) of clans in the Atlantic and Pacific (data from Figure 6.2 of [62], in which the codas from Atlantic and Pacific clans are combined in one analysis). Note: the identity codas of EC1 all contain five clicks and are of the form ‘click-{pause}-click-{pause}-click-click-click’.

When Luke Rendell and I analysed the vocalizations of social units recorded from the waters off the Galápagos Islands we noticed an intriguing pattern. It suggested a phenomenon that has since become the driving force behind much of our thinking about sperm whales, as well as our research at sea. The sperm whale communicative vocalizations that we analysed are called codas. Codas are sequences of sperm whale clicks that differ from echolocation in that they are limited temporally (usually 3–12 clicks per coda), have distinct stereotyped patterns and are made in social contexts [19]. Off the Galápagos in the 1980s and 1990s, the coda repertoire of a unit typically was organized around either of two alternative motifs: ‘click-click-click-click’ (Regular) or ‘click-click-{pause}-click’ (Plus-one) [20] (figure 1). In either case an individual coda could have from 4 to 10 clicks, but for the first set of units the clicks were regularly spaced, and for the second the last click in each coda was preceded by a pause. So, we divided the sperm whales that we studied off the Galápagos into two vocal ‘clans’. What were these clans?

The dialects marked social divisions. The Regular clan social units only formed groups with other Regular social units, and the Plus-ones only with other Plus-ones, even though social units of both clans were often in the same area [20]. Clans had different mitochondrial DNA distributions, but remarkably similar nuclear DNA [13,21,22]. This strongly suggested that a female sperm whale normally remains in the same clan as her mother, but may mate with a male from another clan. The mature males start making trips from their cold water habitats back to the tropics to mate when in their late 20s [17]. Presumably, then, the characteristic dialects are learned from the mother and other females in a young sperm whale's social unit, as well as perhaps from grouped members of other social units, which will be of the same clan.

Primarily using the data from the two Galápagos clans, inter-clan differences appeared in a range of metrics: in how the groups moved, in their feeding success, in their reproductive rates, in their ‘micro-’ distributions around the Galápagos Islands, and in how they arranged babysitting for their calves [23–29]. Some of these distinctions were very strong, others more subtle (table 1). As with the dialects, these differences really had to be the result of social learning, and so forms or results of culture, with culture defined as ‘information or behaviour acquired by social learning shared by members of a community’ [30]. This is similar to most definitions used by scientists studying non-human culture [e.g. 31,32].

**Table 1.** Evidence for behavioural and other differences between sperm whale clans using the same areas.

trait	strength of evidence	references
coda dialect	excellent	[20,33–36,39]
geographical extent	excellent	[20,29,39]
small-scale distributions (10's km)	good	[25,44]
large-scale movements (days-years)	good	[28,29]
small-scale movements (hours)	good	[23,29]
feeding success	good	[23]
changes in feeding success with El Niño	ok	[23]
reproductive rates	ok	[24]
diving synchrony (babysitting)	ok	[26]
homogeneity of social relationships within social units	ok	[26]
duration of social relationships	indication	[26]
diet	indication	[27]

Analysis of acoustic data found evidence of two or more sperm whale clans in the waters off Brazil, Chile, Japan, Mauritius and in the eastern Caribbean [20,33–36]. The clans seemed to be a fundamental part of sperm whale life. Because two or more clans are frequently found in the same area with basically the same environment, and nuclear genes are well mixed between clans, culture is the only tenable explanation for the differences between clans. This gives an advantage over studies of the cultures of many other species in the wild, such as chimpanzees (*Pan troglodytes*), as sperm whale scientists are largely spared the debates about alternative genetic or environmental drivers of behavioural differences [37]. So, sperm whale scientists study clans to understand this extraordinary animal, and to learn about how cultural evolution can work in non-human species.

## 3. Unexpected findings about sperm whale clans

As studies of sperm whale cultural clans rolled slowly in over the past 20 years, so did some unexpected results:

### 3.1. Cultural turnover

There was a hiatus in studies of the Galápagos sperm whales from 2000 to 2012. There were few reports of sperm whales off the Galápagos during that interval. Research resumed in 2013 and revealed quite a surprise. There was no sign of the Regular or Plus-one clans, but two different clans were in residence: the Short clan, members of which had occasionally used Galápagos waters in earlier years, and the Four-plus clan, members of which we had previously found off Chile and in the central Pacific [38]. This was perhaps comparable to returning to Montreal after a 14-year absence, finding it was still largely bilingual, but now Spanish and Urdu had taken over from English and French! We do not know what happened to the original Galápagos occupants.

### 3.2. Size of clans

Taylor Hersh led a recent collaborative analysis of sperm whale recordings from right across the Pacific Ocean, which identified about seven clans [39]. But the Pacific contains roughly 300 000 sperm whales [extrapolating from 11], suggesting an average of about 20 000 females per clan^1^. This is a huge number for culturally defined entities outside modern human ethno-linguistic groups. It also suggests a major exception to Zhou *et al*.'s [40] general rule of a factor of 3 between the numbers of individuals in nested hierarchical levels of human social structures (e.g. military hierarchies), which also roughly applies to baboons (*Theropithecus gelada* and *Papio hamadryas hamadryas*), elephants (*Loxodonta africana*) and killer whales (*Orcinus orca*) [41], but perhaps not to human ethno-linguistic groups [42]. Social units of about 10 sperm whales and groups of about 30 fit the pattern, but our next known level, the clan, has about 20 000 sperms. Are we missing 4–5 intermediate levels of social structure, or are sperm whale clans fundamentally different from hierarchical social levels of other social mammals?

### 3.3. Spatial spread and size of clans

The seven clans in Hersh *et al*.'s study have very different ranges. One, the Short clan, was found right across the Pacific, from Japan to Chile (spanning about 10 000 km) whereas the Plus-one clan was only heard off the Galápagos and mainland Ecuador (about 1000 km apart) [39]. Presumably the numerical sizes of the clans vary accordingly, with the Plus-one clan containing considerably fewer than my rough average of 20 000 animals (see above), and the Short clan many more.

### 3.4. Fundamentals of dialect distinctions: motif versus single identity coda

Each clan identified to date makes some coda patterns that are rarely heard from other clans, which we call identity codas, and other coda patterns that are common to different clans, non-identity codas [43]. During their detailed studies of sperm whales off the island of Dominica in the Caribbean, Shane Gero and colleagues recognized that the social units whom they knew came from two clans [33]. EC1 included all the social units that they had documented with the exception of two social units, who were occasional visitors to Dominica and made the quite distinct EC2 repertoire. However, unlike the Pacific clans, each of which can make several identity codas with a varied number of clicks but with a distinctive motif—such as the Plus-one clan with its pause before the final click of a coda, or the Palindrome clan whose codas often sound similar when reversed—EC1 and EC2 have distinctive single identity coda patterns with a fixed number of clicks (five) that they make much of the time: ‘click-{pause}-click-{pause}-click-click-click’ (EC1) and ‘click-{pause}-click-{pause}-click-{pause}-click-{pause}-click’ (EC2) (figure 1) [33,39].

### 3.5. Clan scales and oceans

Felicia Vachon extended Gero's research to the waters off the other islands of the Lesser Antilles, about 300 km north and south of Dominica [29]. As this span was less than the range of even the least extensive clan in the Pacific, she expected to find the Dominica social units right along the island chain, with EC1 predominating. But it was not like that at all. The known Dominica whales were rarely found far from Dominica, and different islands had either very predominantly EC1 or very predominantly EC2 social units [29]. Differences in distribution between the clans could not be explained by differences in habitat features, but rather the vicinity of clans to particular islands themselves (most likely caused by the traditions of social units, closely linked to their clan membership) [44]. As a dramatic example, the waters off Martinique, just 30 km from EC1-dominated Dominica, contain almost entirely EC2 social units. The scales of movement and habitat use by the different clans are over an order of magnitude smaller off the Lesser Antilles compared with the Pacific [29].

### 3.6. The strange case of EC3

During her surveys of the Caribbean, each of the social units that Vachon encountered was a member of either the EC1 or EC2 clans, except one [29]. This unit largely made codas containing 9–11 regularly spaced clicks, a repertoire entirely distinct from those of the EC1 and EC2 social units (figure 1). It was encountered six times over the 2 years of surveys, always in the EC2 waters of Martinique and St Lucia, and on one of these occasions interacted with an EC2 unit. Is this unit from an entirely different clan (EC3?) with little aversion to EC2, or is it a vocally idiosyncratic part of EC2, or something else?

### 3.7. Symbolic marking

Symbolic markers denote cultural group identity to individuals inside and outside of a cultural group, and are used to delineate social relationships [45]. In their mapping of Pacific clans, Hersh and colleagues looked at dialect similarities between clans [39]. They did this both for the identity codas, made primarily by just one clan, as well as non-identity codas which are more generally shared among clans. They found that the similarity in use of identity codas (but not non-identity codas) was lowest for pairs of clans that shared habitat. This is what would be expected if clans that share habitat experience pressures to sharpen the distinctions between their cultural expressions, which both modelling [45] and empirical studies of humans [e.g. 46] show to be an expected outcome of symbolic marking of group membership.

## 4. Sperm whale clans and human societies: parallels and contrasts

During the discovery and investigation of the sperm whales' clans, we considered potential models and parallels. The rich vocal and social lives of birds have parallels in Cetacea, for instance the songs of passerines and baleen whales [47], but we know of nothing avian much like the sperm whale clans. Killer whales are closest, and naming the structures ‘clans’ followed the killer whale terminology [20]. But killer whale cultural groupings are either on a much smaller scale (hundreds to a few thousand whales in each ‘community’, and even fewer in a killer whale ‘clan’), or, at the larger scale of ‘ecotypes’, much more ecologically and genetically distinct from one another, so potential subspecies [48].

Ethno-linguistic groups of humans (groups of individuals that share both perceived attributes and language) are an attractive model as we consider sperm whale clans [39]. These groups are a prominent element of human society, in particular by setting boundaries for social relationships and cooperation, but they are not always easily defined or delineated [49]. They vary greatly in scale, covering a median area of about 10 000 km^2^ (ranging between 10 and 10 000 000 km^2^) [49], and so spanning a median of very approximately 100 km and up to about 2000 km. The population sizes of ethno-linguistic groups vary from a few hundred for hunter–gatherers to millions for modern nation-based peoples.

Thus, sperm whale clans and human ethno-linguistic groups share large scales both numerically and in geographical extent, distinctive behaviours, and social barriers. The scales are such that members of a sperm clan or human ethno-linguistic group may never encounter most other members of the same clan/group. However, if they did encounter them they would usually recognize such individuals as clan/group co-members from distinctive culturally-inherited group-specific behaviour. When humans are the comparison species, it is important to steer a path between unthinking anthropomorphism (assigning human characteristics to non-humans) and anthropodenial (denying human properties to other animals) [50], but fortunately in this case it seems a fairly wide path.

Hersh and colleagues noted that the distributional patterns of sperm whale clans in the Pacific resemble those of human ethnolinguistic groups [39]. Spurred by the suggestion that symbolic marking of cultural groups is uniquely human [51], they used results from humans and predictions from models that cultural groups are most marked at boundaries to examine the hypothesis that sperm whales use identity codas as symbolic markers, and the data supported the symbolic marking hypothesis. We can undoubtedly go further in using knowledge of human societies to generate hypotheses. But we need to be careful that they make sense in the sperm whale context.

However, there are fundamental differences between human ethno-linguistic groups and sperm whale clans. Only about 12 cultural attributes have so far been found to differ among sperm whale clans (table 1), whereas human ethnolinguistic groups differ in countless documented and undocumented ways. The time scales also seem radically different, with human ethno-linguistic groups having (necessarily, by definition) arisen since the origin of language about 150 000–350 000 years ago [52], while sperm whales evolved about 24 million years ago [8], and clans may have been around for much of that period. Cooperative care of infants is a key attribute of the societies of both sperm whales and humans. In sperm whales this cooperation is primarily among bonded females who are largely matrilineally related [15]. By contrast, in most (but not all) modern human societies, the pair bond between male and female is key to raising children. However, Lesley Newson and Peter Richerson argue that collaborative care among females, as in sperm whales, was a key element of the social evolution of humans [3].

## 5. Considering sperm whale clans; lessons from human history

Understanding of humanity's (pre-)history has been formed by worldwide academic effort in archaeology, anthropology and other disciplines over many decades. By contrast, there are perhaps a dozen scientists who think much about sperm whale clans, and only in the last 20 years. Discoveries about the processes of human prehistory may be informative as we try to document and understand the clans of sperm whales. For example:

### 5.1. Resources and society

Biologists typically see resource acquisition as a major driver of evolution. For instance, the remarkable division of killer whales into culturally-distinct ecotypes is believed to result from cultural resource specialization [48,53]. The traditional assumption that agriculture drove the increased complexity of human societies [e.g. 54] is wrong as a universal process: in prehistory, agriculture had a very complex relationship with social organization [4]. We should not necessarily expect ecology to have driven the evolution of distinct clans, or for sperm whale cultural clans to differ much ecologically. This is suggested by the results of Marcoux *et al*. [27] who found only small differences between the stable isotope signatures of members of different clans in the same areas of the South Pacific.

### 5.2. Variability

There has been and is enormous variability in the nature and structure of human cultural groups as well as how they interact with one another, both spatially and with time [4]. We should expect the same with clans. There is some evidence for this, at least in the spatial domain. Hersh and colleagues' study indicates large-scale processes working across the Pacific [39], but because female sperm whales are largely limited to tropical and subtropical waters, the Atlantic and Pacific have little connection for them, and our results to date suggest that social and cultural arrangements, including the use of space, are quite different in the two ocean basins [29,39]. This gives a situation comparable to the independent development of human societies in Eurasia-Africa and the Americas: two, or perhaps three (if North and South America are separated for humans, and the Indian Ocean is distinct for sperm whales), replicates for each species. And then there is the Mediterranean whose small population of sperm whales is largely discrete, somewhat comparable to a human population on a fairly isolated island (Australia?). Shane Gero is leading a worldwide comparison of the coda dialects of sperm whale clans, which is likely to have most interesting, and unexpected, results. Information on temporal variation in clan behaviours and structures is less accessible than variation with space. The longest field studies have lasted 3–4 decades, barely two generations for sperm whales, and there is no archaeological record for oceanic mammals. However, genetic correlates can potentially provide indications of clan membership dynamics [21] as well as the evolutionary record of culturally-transmitted behaviour (e.g. [55] for killer whales), as might an evolutionary analysis of coda structure.

### 5.3. Vocalizations and behaviour

It turns out that ‘ethno-linguistic’ is not a very good descriptor for at least some human groups. Both language and other important and variable human behaviours (such as foraging methods, decorative styles and religious practices) are largely culturally transmitted (i.e. socially learned). However, for various reasons, including some behaviour being largely transmitted matrilineally and some patrilineally, the ethnic and linguistic components do not always match too well [4]. Human groups may have similar languages but very different means of sustenance or political systems, or vice versa [4]. Recent examples of this uncoupling include Protestants and Catholics in Northern Ireland both speaking English, or Ashkenazi Jews speaking different native languages in different European countries. Shane Gero has asked me several times if we should call sperm whale clans just ‘clans’ or ‘coda clans’. Most clans we only know from their coda dialects; information on other clan characteristics is often unavailable (see above). For the Regular and Plus-one clans studied off the Galápagos between 1985–1995, we know that the vocal differences map on to major distinctions in other behaviour, and the results of other behaviour [23–26]. There is also a little information on clan-distinctive behaviour for clans off Chile in 2000, and in the Caribbean in 2019–2020 [23,29]. So we have made the assumption, largely based on those two Galápagos clans, that distinctive behavioural practices (cf. ‘ethno‘) and dialects (cf. ‘linguistic’) largely line up, and that we can use ‘clans’ to include both. Maybe not…

### 5.4. Gender

In some human societies male behaviour principally drives societal organization, leadership and innovation; in others female behaviour is generally more significant; and there are many societies in which the genders have similar or complementary roles [4,56]. With sperm whales the assumption is that society and cultural transmission is almost entirely female based [see 57]. A breeding male attends a social unit for a matter of hours or less [58], and it has been tacitly assumed that the only important transfer is of sperm. Perhaps not…

### 5.5. Schismogenesis

Schismogenesis is the hypothesis that humans, individually or as groups, define themselves against one another, and so interacting groups deliberately adopt strongly contrasting behaviour [59]. The outcomes resemble the distinctions expected from symbolic marking—behavioural differences between interacting groups—but they can be much broader and deeper: holding slaves or not, having an autocratic or democratic political system, etc [4]. Perhaps the very dramatic difference between the movements of the Regular and Plus-one clans off the Galápagos [23] has some schismogenesis at its root.

### 5.6. Consensus decision making

One of the more controversial contentions of ‘The Dawn of Everything’ is that throughout history and prehistory and in a wide range of circumstances, some groups of humans have used consensus, truly democratic discussions, rather than leadership from ‘the top’, to make important decisions [4]. Sperm whales, sometimes travelling in very large groups, make important communal decisions about where to go, how fast to travel, and when to feed in an environment varying substantially in resources and containing lethal predators. Many of these changes in communal behaviour are slow and messy—a group of sperm whales can take an hour or more to make a 90^o^ turn—so likely democratic [60].

## 6. Implications for human social evolution

The existence of large-scale complex clans of sperm whales has potential implications in the search for drivers of human social complexity. Many such drivers have been suggested including bipedalism, mobility, cooperation, brain size and/or complexity, syntactic language, monogamy, control of fire, tool-making, opposable digits and the capacity for culture itself [e.g. 61]. Some of these drivers can now be ruled out as *necessary* conditions for the development of the large-scale, culturally distinctive social structures whose members experience within-group symbolically marked identity, that humans share with sperm whales (e.g. bipedalism, monogamy, control of fire, tool-making, syntactic language and opposable digits). In both species the evolution of such large-scale structures was likely complex and convoluted, but interspecific comparison indicates that shared attributes, such as large and complex brains, cooperative behaviour, mobility, or cultural capacity, may have been important factors.

## 7. Conclusion

Sperm whales are *very* different from humans, and sperm whale clans are *very* different from human ethno-linguistic groups. It seems exceedingly unlikely that sperm whale clans have more than a small fraction of the richness and complexity that distinguish human groups. But there are some broad-scale similarities that have few parallels elsewhere.

Sperm whale scientists can take advantage of these parallels, and of all the research and thought that has gone into documenting and understanding human societies, as we try to build a picture of the nature of a most unusual animal. For instance, the dynamics of human societies may suggest possible processes that could drive clan distinctions (e.g. schismogenesis), and warn against generalist assumptions (e.g. a clear relationship between clan distinctions and ecology, and that clans are entirely about females). The comparisons may also be of importance as we consider the evolution of large-scale structures in human societies. That similarly large-scale structures exist in another species provides support for some generative factors and against others.

## Data Availability

This article has no additional data.

## Footnotes

1 With an estimate of 150 000 whales in the North Pacific, which is about half the sea area of the Pacific, and half the animals being males, we get about 150 000 females, and so an average of roughly 20 000 animals in each of seven clans. These numbers are very approximate.
